# Metaphorical Description and Model Description of Complex Systems

**DOI:** 10.1155/2022/3094010

**Published:** 2022-07-07

**Authors:** Huaxin Huang, Yuhang Li

**Affiliations:** Center for the Study of Language and Cognition, Zhejiang University, Hangzhou 310028, Zhejiang, China

## Abstract

From metaphorical description to model description is the symbol of the maturity of complexity science. This paper focuses on two model description methods of complex systems: formal system model and relevance theory model, and compares their advantages and disadvantages. These two models are descriptions of complex systems from different perspectives and have important and irreplaceable significance in the theoretical research and practical application of complex systems. The future research direction of complex systems is not the “integration” of the two models but the alternation of the two models, which scientifically reveals the essence of complex systems from multiple perspectives. In view of the complexity of education and the scientificity of educational evaluation, a fuzzy evaluation method is proposed. Fuzzy evaluation is a mathematical thinking mode based on fuzzy theory and mathematical methods, which studies fuzzy and complex phenomena, and provides quantitative relationship and description for educational evaluation.

## 1. Introduction

Complexity science studies complexity and complex systems, emphasizing, understanding, and grasping the research object according to the true face of things. Complex systems have the characteristics of nonlinearity, chaos and fractal, fluctuation and mutation, randomness and contingency, organization and self-organization, adaptability, dynamics, and so on. Education is a special complex system, which generally has the characteristics of nonlinearity, irreversibility, self-organization, indeterminacy, contingency, and imbalance. With the development of complexity science and the deepening of people's understanding of the complexity of educational phenomena, it provides a new direction for guiding educational evaluation [1].

At present, apart from the complex dynamics theory represented by Cameron et al., there are few studies that regard metaphor as a system with complex attributes and try to model it. Only a few studies such as Gongyumiao's (2018) “relational category model” have been discussed. As for the concretization of metaphor, although Lakoff and others' views on the concretization of metaphor have been widely influenced, most studies focus on the review and interpretation of the concretization of metaphor, or the analysis of some concretization features in the cognitive process of metaphor, and few studies have conducted specific modeling of metaphor from the perspective of concretization.

Metaphor is an important observation object in linguistic research and a language carrier of emotion and value judgment. In recent years, linguists represented by Cameron, Gibbs, Deignan, and Semino have introduced the complex system theory of natural science into the study of metaphorical language, and proposed a discourse dynamics approach to metaphor. The research results were published in top linguistic journals, which brought about a complex systematic turn in metaphor research and broke the long-term control of conceptual metaphor theory (CMT) over metaphor research [2–4]. CST-based metaphor research focuses on the systematic use of metaphor and puts forward the following assumptions: metaphorical language is not only the product of image schema but also driven by discourse purpose, social context, language context, and cognitive activities. Metaphorical language in discourse events constitutes a complex cross system, interconnection, and open development [5–7].

## 2. Complexity of Education

In the field of educational science, the object of educational research is complex. When man is the subject and object of research, man's thinking, emotion, will, and behavior not only have various characteristics of the natural material world but also have subjective initiative. Its essence is the meaning world and value world, which can be truly grasped only through understanding. What educational research faces is not a pure factual world but a cultural world. The core of the cultural world is value and significance, which has strong individuality, diversity, acquisition, and contingency. Therefore, educational research cannot avoid judging and evaluating its value and significance. For example, the teacher-student relationship of the interactive subject is a process of meaning and value construction for both teachers and students [8].

The educational system is complex. First, the people who receive education are complex. As the object of education, people synchronize with social development, affect social development, and constitute the sum of society through education. Human social development is in the process of contradiction, with fuzziness, spiral development, and certain unknowability. Secondly, the development of education is subject to the complexity of social system. The development of social politics, economy, and culture directly affects the development of education and determines the starting point and upper limit of educational development. Human education is to serve human life and production from the beginning. After human society enters the class society, education also has relevant attributes, such as politics. The basic value, purpose, content, and educators of education are determined by the ruling class. The freedom and autonomy of educational practitioners and theorists can only be within the scope of the system. In modern society, in a society with a high degree of human civilization, education is still affected by politics, but in order to meet the objective needs of the educated, it also has the corresponding marketability, so that educational activities are affected by the economic market and the supply and demand of education. Thirdly, the educational effect is reflected in the growth and development of educational objects. Human growth is a complex process, which cannot be measured by a single method. Therefore, educational evaluation is also complex. Scientific evaluation is very helpful to the development of education. Among them, scientific evaluation based on mathematical thinking and mode is easier in natural disciplines, but it is extremely difficult in pedagogy. [9] Scientific educational evaluation is aimed at some quantifiable and descriptive educational phenomena, while fuzzy, nonlinear, and unpredictable are difficult to evaluate. For example, it is difficult to make an accurate evaluation of the current new curriculum reform. The evaluation should not only follow the history but also be disturbed by the influence of existing methods and thinking set, and be unable to do anything about the new methods.

## 3. Metaphors as Complex Systems

**Figure 1:**
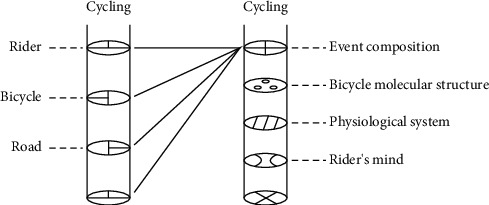
”Cycling.”

The guidance of the complex system view of metaphor to the empirical research is as follows: (1) complexity emphasizes the comprehensive connection of metaphorical language, language sender, and social culture, which can provide a multiangle analysis topic for the empirical research of metaphorical language; (2) dynamism helps to describe the difference between the initial state and the changing state, and can provide an observation population for investigating the development and change of metaphorical language; (3) nonlinearity requires the study to describe the metaphorical language form in a diachronic and retrospective way, which is conducive to improving the scientificity of the analysis process. Therefore, highlighting complexity, highlighting dynamics, and being loyal to backtracking are the methodological principles of empirical research called for by the complex system view of metaphor [10]. Reference [11] once put forward that metaphor research in the 21st century should “take metaphor out of the mind and put it into the real cultural world”. Compared with the classical metaphor theory, the complex system view of metaphor is more conducive for researchers to complete this task. The overall meaning of a metaphor is not the sum of its parts; the meaning elements are in a nonlinear relationship with each other. The meaning elements arising from different cognitive bases are at different levels and dynamically interact with each other across levels to generate the overall meaning of the metaphor. As a system of nonlinear interaction, some processes in the system are dramatic and steep, with a self-amplifying mechanism of change. The drastic, rapid, and massive changes prompt the emergence of more abstract meanings. The neglect of complexity is one of the reasons why the system represented by the mapping theory model fails in some scenarios [12]. Decomposing metaphorical meaning into simple attribute elements or frame elements, the elements can only grow in meaning through linear superposition, while the actual meaning building process of metaphor can often accomplish a qualitative leap in nonlinear causality between elements to achieve an ordered and more than expected meaningful whole. We try to show the difference between a simple system and a complex system as follows:

The left side of Figure 1 represents a simple system, the elliptical cross section of each layer represents the decomposed parts of the system, the inner edges of each decomposed part overlap roughly, and the system's wholeness can be restored by simple summation.

On the right side of the diagram, the elliptical cross-sections at each level represent complex systems, and the inner edges of the decomposition parts represented by the elliptical cross-sections at each level rarely overlap, and some decomposition parts may even be completely different from each other. The elements in each decomposition part cannot be restored to the wholeness of the system by simple summation.

The meaning that can be constructed on the right side of Figure 1 may only be a part or even a small part of the left side of Figure 1. Therefore, metaphorical models that lack a complex system perspective setting tend to construct inadequate metaphorical meanings. For example, a metaphorical expression can be constructed for the above events as follows:

### 3.1. Love is Cycling

If you look carefully, there are naturally certain structural similarities between falling in love and cycling. For example:

However, it is not difficult to imagine the following case.

### 3.2. A Rode a Bike in an Open Area Last Week and Felt Very Fresh and Comfortable on that Ride

Then, he fell in love for the first time in his life. (The intention is to express that it is very refreshing and comfortable to fall in love)

A: Falling in love is riding a bicycle.

Example (2) meaning construction is not after abstract mapping between conceptual frames, but conveys information about the subject's bodily sensations, emotions, etc. It is necessary to include this part of the transmission, which is different from the mapping between abstract structures, as a relatively independent module in the metaphorical model, allowing the model to be handled differently.

In addition, the metaphorical meaning construction process also has complex system properties such as self-organization, vagueness, and uncertainty, e.g., the logical vagueness of metaphors is consistent with the basic tone of complex systems against binary logic emphasizing intermediate states. Due to the limitation of space, it is not possible to elaborate on each of them in this paper. In conclusion, complexity science has changed the traditional simplistic, linear, and static way of thinking, and provided a systematic perspective for studying the process of metaphorical meaning construction.

## 4. The Proposed Mental Space Model of Embodied Embedded Metaphor

### 4.1. The Architectural Basis of the Model - The General Composition of the Linguistic-Psychological Space

Since the inherent cognitive mechanism of metaphor is a complex system, the model of metaphorical meaning construction must also follow the basic architecture of a complex system. The basic architecture of a complex system is characterized by multiple levels and interrelationships between levels. By looking into the metaphors, we can find this multilayered structure of interrelationship in the mental space. In particular, the system of psychological space theory developed by Lacan, Kristeva, and others from Freud's view of consciousness. Lacan set up the psychological cognition of human subjects as an imaginary space containing emotions, desires, etc., and a symbolic space containing logic, social statutes, etc. Kristeva, on the other hand, sets up mental space as presymbolic space and symbolic space, and relates the two as a dynamic system [13]. This open mechanism that transcends linguistic forms constitutes a dynamic system in which different psychological space areas interconnect and interrelate.

The insights of Lacan, Kristeva, and others on mental space are largely in line with the insights of brain science on consciousness. Consciousness can be divided into two broad categories: primordial and higher consciousness [14] or core and extended consciousness [15]. In general, primordial consciousness is the “here and now” consciousness; extended consciousness is the “not here and now” consciousness that extends from primordial consciousness.

The original consciousness and the extended consciousness, the presymbolic space and the symbolic space are not independent bodies that do not interfere with each other but constitute a layered and interrelated linguistic psychological space that has the properties of a complex system, which also serves as the architectural basis of the embedded metaphorical psychological space model of the present stationery itself.

### 4.2. The Embedding of “Virtual Body”

In order to investigate the cognitive mechanism in the process of metaphor generation, application, and understanding, it is necessary to analyze the metaphor from the perspective of the experience and perception of linguistic psychological space.

Space is the place where meaning is produced, where the issues involved are the production of meaning and perception, and the construction-deconstruction of it [16]. The medium is the “body” that runs through the objective world and the psychological space. If the embodied view of metaphor is recognized, then metaphorical perception is based on the new perceptual space created by the subject through mental simulation, imagination, virtual perception, etc., and its integration [17]. This “body” is not only the body that we can touch in the objective world but also the “virtual body” in the mental space. The “virtual body” is the coordinate for experiencing and perceiving the mental space. It is because of the existence of the “virtual body” that the subject can experience and perceive in the mental space, and thus dynamically generate meaning. The “embodied embeddedness” advocated in this paper refers to the process of metaphorical meaning construction in which the “virtual body” or “virtual me” is embedded in the psychological space, assimilating itself into the space as a person, creature, or even a person in the space. The process of embodied embedding refers to the process of embedding the “virtual body” or “virtual me” into the mental space, assimilating oneself into a person, creature or even inorganic object in the space, setting up a specific perspective, perceiving the environment in the space, and thus acquiring a large and complex meaning of feelings, emotions, logic, values, and knowledge related to the “world” presented in the mental space. The basis of this process is embodied embedded cognition [18–20]. Embodied embedded cognition integrates the emphasis of embodied cognition on the role of the body with the emphasis of embedded cognition on the subject's use of the environment to accomplish part of a complex cognitive task. This cognitive mechanism allows the subject to use the “virtual body” as a coordinate in the metaphorical cognition process and to rely on sensory and motor abilities to cognize the environment in order to complete subsequent complex cognitive tasks.

### 4.3. Embodied Embedded Metaphorical Mental Space Model

**Figure 2:**
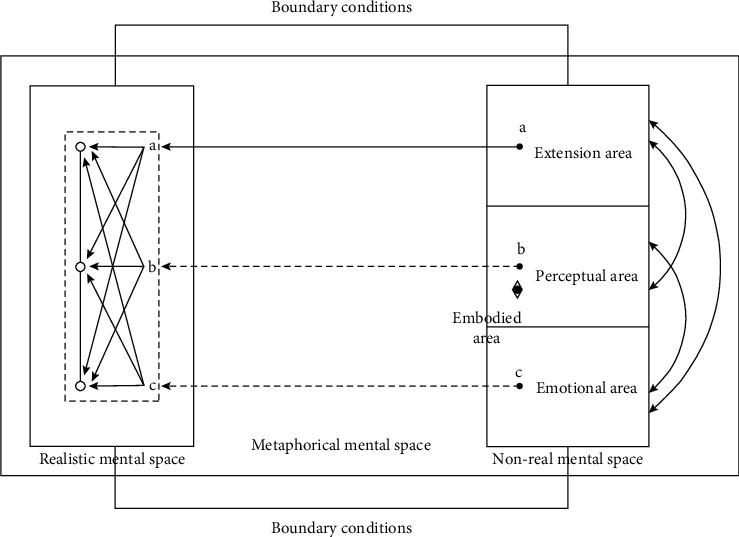
Embodied embedded metaphorical mental space model.

Based on the embodied embedding of the subject in the mental space and the hierarchical interrelation of the mental space, this paper tries to propose a model of embodied embedded metaphorical mental space. The model is as follows:

The model considers that the basic framework of metaphorical mental space consists of real mental space and nonreal mental space. Each space is roughly divided into embodied and extended areas, and the embodied area is divided into perceptual and emotional areas. The subject activates the real psychological space and the nonreal psychological space with external stimuli such as verbal symbols, and embeds the “virtual body” into the nonreal psychological space to set up the perspective, which corresponds to the eye symbol on the right side of Figure 2. The “I” as the subject (including the “I” as the observer) perceives the environment and the self in the nonreal space, and obtains various elements of body sensation, emotion, evaluation, logic at different levels. Therefore, a slight change in the content of the mental space means a change in the environment perceived by the virtual subject, and causes a change in the overall meaning of the result of this multilevel interaction, just like the “butterfly effect.” The nonreal mental space is mapped to the real mental space, and the way of mapping is not simply homogeneous but varies according to the level of the elements. The main mapping methods are analogical mapping and simulation mapping. Each meaning element is self-organized under the constraints of boundary conditions to compete and cooperate, and finally integrated into a metaphorical meaning as a whole structure (as shown in Figure 2 left).

The main ideas and specific implications about the model will be discussed in detail in the next section.

## 5. Main Ideas and Specific Connotations of the Embodied Embedded Metaphorical Mental Space Model

### 5.1. Composition of Metaphorical Psychological Space

Although Edelman, Damasio, and others have divided consciousness into two main levels, they also emphasize the distinction between “self” and “nonself” in primordial consciousness. For example, Damasio (2010) distinguishes between the protoself, the core self, and the biological self, to a certain extent distinguishing the consciousness bound to the body from the consciousness generated by the protoself after encountering the outside world. Based on this distinction between the consciousness and the self in the encounter between the body and the outside world, we set up the metaphorical psychological space into the embodied area and the extended area, and set up the perceptual area and the emotional area in the embodied area, based on the dichotomous system of the linguistic psychological space of Lacan and Kristeva. This constitutes a pseudotriple structure that starts from the embodied area and is unified in the interaction between levels, covering physical, perceptual, and cultural aspects. The reason for starting with embodied areas rather than perceptual areas is that metaphor understanding is based on emotions generated by the interaction between the subject's own experience and the context [21], and emotional activation activates and penetrates the bodily basis of metaphor understanding, thus facilitating the subject's faster understanding of metaphorical meaning [22]. It can be argued that embodied areas jointly initiate the “online” construction of metaphorical meaning, with perceptual areas playing only a partial role.

Specifically, the perceptual area mainly includes bodily sensory elements such as five senses, spatiotemporal sensations, and body movements; the affective area mainly includes emotional elements such as emotions and evaluations; the extension area mainly includes the knowledge system based on the information obtained from experiences and perceptions. These elements are interrelated across levels, e.g., the “bicycle” obtained in the perceptual area of the nonreal mental space in example (2) can trigger the event framework and reasoning related to the bicycle, which is obviously an element of the extended area; the “face touch while riding a bicycle” obtained in the perceptual area can trigger the “pleasure.” “This is clearly an element of the affective region. In Figure 2, we show the nonlinear interconnections between the regions by the two-way arrows between the different regions on the right side of the figure.

Another difference between the composition of the mental space of metaphors and the mental space of language in general is the simultaneous establishment of “reality” and “unreality.” The subject knows that the binary logical value of the metaphor is “false” when he generates, uses, and understands it. This reflects the logical fuzziness of metaphors. Fuzzy logic emphasizes that “A” and “not-A” are not binary contradictions, but nonbinary oppositions that can be established simultaneously, with a range of other possibilities in between. According to fuzzy logic, the propositions “falling in love is riding a bicycle” and “falling in love is not riding a bicycle” in example (1) and example (2) can be established at the same time. In the process of metaphorical meaning construction, this simultaneous establishment of “A” and “non-A″ also implies that there can be “reality” and “nonreality” in the mental space that the metaphor may activate. The simultaneous establishment of “reality” and “nonreality” in the metaphorical meaning construction process also implies that the mental space that the metaphor may activate can also exist. In other words, there are two ambiguous and contradictory spaces in the metaphorical psychological space: one is the real psychological space and the other is the nonreal psychological space. The activation of the real psychological space depends on the perception of reality, for example, the cognitive subject's perception of the real situation at the moment of receiving the metaphor “falling in love is riding a bicycle.” The activation of the nonreal mental space is based on imagination and the awareness of “knowing falsehood as falsehood.” For example, the perception of the metaphor “falling in love is riding a bicycle” in which “riding a bicycle” is not really talking about “riding a bicycle.”

### 5.2. Two Different Metaphorical Mapping Mechanisms

#### 5.2.1. Analogical Mapping

The physiological basis of human beings determines that there can only be one “body” that can participate in sensation, so embodied embedded experience and perception can only take place in nonreal space first, and there is a psychological distance and barrier between real space and nonreal space. However, this distance and obstacle does not prevent the extension of the area including logic and social cognition. Because thinking and symbolism are not limited to the immediate world.

According to the semantic theory of possible worlds, the real world is one of many possible worlds. Possible worlds are worlds consisting of clusters of propositions that do not actually occur, but exist in the subject's mind and are connected to the real world in some way. A common mechanism is known as analogical referencing mapping [23–25], and the correspondence between equivalents in different worlds relies on this cognitive mechanism.

From the perspective of “possibility,” the nonreal mental space undoubtedly also carries a possible world. However, for the “virtual body,” the nonreal mental space also carries an “immediate” world. That is to say, due to the embodied embedding model, the real mental space and the nonreal mental space are “possible” to each other, including the extended consciousness of logic and social cognition, which can transcend reality and nonreality. This determines that the equivalence between the extension area of real psychological space and the extension area of nonreal psychological space can be corresponded by analogical mapping mechanism. This is one of the mapping mechanisms that integrate the metaphorical real mental space and nonreal mental space.

#### 5.2.2. Emotional Mapping

Another mapping mechanism is a cognitive process that cannot be called analogous. As mentioned above, embodied embedded experience and perception cannot occur simultaneously in real and nonreal mental space, and there is no equivalent analogical mapping mechanism between the embodied area in nonphysical mental space and the embodied area in real mental space. We believe that the mapping between the two is based on simulation. Embodied cognition, whether sensory or emotional, can be transmitted from other to self or from unreality to reality through simulation. Its physiological basis lies in neural simulation mechanisms. Brain science research, represented by the study of mirror neurons, has revealed the physiological mechanism of the mental activity of “imagination” - simulation. Even if there is no real movement or sensation in the real world, when a subject perceives a specific object or perceives another person performing a relevant behavior, the same brain regions as the real movement or sensation may be activated.

We believe that the mechanism of simulation is particularly important in the process of constructing metaphorical meanings premised on nonreal imagery. The elements in the embodied area of the nonreal mental space cannot be mapped to the real mental space through analogical correspondence as the conceptual elements in the extended area, and these embodied elements are indispensable to the meaning construction process of many metaphors. If the meaning construction of metaphor only covers the analogical mapping process, its value will be greatly reduced, and it will not be much different from the so-called “analogy.” By embedding the “virtual body” in the nonreal mental space and perceiving the imaginary environment from a specific perspective, the “virtual body” will gain five senses, emotions, and other experiences. These experiences in the nonreal mental space activate the same brain regions in the real body through simulation, thus achieving the same embodied elements in the real mental space. This “acquisition” is a “reproduction” that transcends analogical equivalence. In Layman's terms, the embodied experience of the “virtual” “I” in the nonreal world can be reproduced in the “I” body in the real world through simulation. This is one of the reasons why metaphors sometimes produce a sense of “vivid imagery.” We refer to this mapping mechanism as a simulation mapping, to distinguish it from an analogical mapping. In the model of Figure 2, the solid arrows between the two mental spaces indicate analogical mapping, and the dashed arrows indicate mimetic mapping, with the apostrophe letters indicating the analogical equivalence of the elements before and after the mapping, and the same letters indicating the reproduction of the elements before and after the mapping.

### 5.3. Self-Organized Competition and Synergy of Metaphorical Meaning

The process of metaphorical meaning construction is not only the process of mapping but more importantly how the metaphorical mental space changes from disorder to order. The cognitive outcome of metaphor is the emergence of a holistic and ordered meaning. We believe that explaining this process requires the introduction of Self-organizing Theory (SOST). Self-organizing theory is an important component of complexity science. The theory assumes that under certain boundary conditions, a system can form a certain structure or function within itself without instructions according to specific rules. Systems that can generate self-organization must satisfy the following three conditions: open systems, nonlinear interactions between subsystems, and energy differences. Regarding the first two, they have been discussed several times above. Regarding the latter, the process of metaphorical meaning construction inherently has unbalanced energy differences between elements, which come from differences in the subject's bodily experience and knowledge system.

It is assumed that the metaphor activates a mental space in which the subject's physical experience gives the highest energy to “tension.” The elements that fit with the “tension” will attract the elements related to it to converge with greater capacity, while other elements are mostly obscured. This process is repeated over and over again, eventually creating an overall structure of meaning. The attraction between elements is a cross-level self-amplifying mechanism, which is mainly reflected in the cross-triggering and self-triggering of activation chains, i.e. two nodes in different activation chains can trigger each other's activation or a node in a certain activation chain is exactly what it needs to be triggered by itself. Take two possible activation chains in the metaphorical mental space of “work is like playing basketball” as an example.

  CHAIN1: C1n1 basketball-C1n2 dribbling-C1n3 sweat-C1n4 body temperature rising
  CHAIN2: C2n1 opponent-C2n2 breakthrough-C2n3 entanglement

We use the code Cxny to represent the yth node on the xth activation chain; then, in addition to the triggers that C1 and C2 each have, C1 and C2 have at least the following cross triggers between them: C2n2-C1n3; C2n2-C1n4; C2n3-C1n3 C2n3-C1n4. The following self-triggering may also exist within C2: C2n3-C2n2.

These activated elements can be used as a response to the meaning of “tension,” which can strengthen the meaning of “tension.” Each trigger implies not only the activation of the relevant element itself but also the growth of the energy of the “tension,” which is equivalent to the metaphorical system constantly reproducing and amplifying the “tension” itself, so that it qualitatively emerges as a new “tension.” All the unobscured elements act spontaneously and collaboratively to achieve the overall order without external instructions, building an overall meaning structure with the meaning of “tension” as the core but more than the meaning of “tension.”

In addition, the noncore meaning of the metaphor may become a secondary local structure after being integrated into the overall meaning structure, in addition to synergistically enhancing the energy of the core meaning. As in example (4), the nonreal situation activated in the mental space may contain the following localities:

  Part 1: the “*I*” as a perceiver confronts the opponents face to face
  Part 2: many people in the audience are cheering, and their voices are heard in the arena
  Part 3: the referee's whistle is sounded from time to time

All the above three parts can activate the “tension” meaning as the core meaning, and most of the other elements in the emotional, perceptual, and extension areas triggered by the chain can also respond to and strengthen the energy of the “tension” meaning, such as the first part can be obtained by analogical mapping. For example, the first localization may obtain the meaning of “work needs to face difficulties” through analogical mapping; the third localization may obtain the meaning of “work has time limitation” through analogical mapping. Although both of these meanings will strengthen the energy of “tension” and eventually integrate into the overall meaning structure, they can still play the value in the form of secondary local structure after integration.

We show the self-organization process in the dashed box in the model schematic, with the circles connected by solid lines representing the overall meaning of the metaphor and the individual circles representing the local meaning structure with their own weights. The whole process in the dashed box shows that the elements obtained from the nonrealistic mental space compete and collaborate across levels to obtain different weights, forming an ordered structure with interrelated and unequal localities, in which the localities with the largest weights become the core of the overall structure.

## 6. Conclusion

In view of the incompleteness of existing metaphor theories and models in explaining related phenomena and their failure in practical application, this paper attempts to describe the cognitive mechanism of metaphorical meaning construction in detail in the context of complexity science and the second generation cognitive science. In the context of complexity science and the second generation cognitive science, this paper attempts to describe in detail the cognitive mechanism of the process of metaphorical meaning construction and build a metaphor model with wider interpretation range and stronger operability. The construction process of metaphorical meaning is a complex system with multilevel interrelationship and interaction. Fuzzy evaluation is a kind of mathematical thinking mode that uses mathematical methods to study and deal with fuzzy phenomena. It can decompose complex educational problems into problem domains with both clear fragments and unified combinations, and can objectively and digitally evaluate and describe the whole and part of educational problems. Today, it has been applied in various disciplines, of course, it has also been cited by the humanities. Naturally, using this method to study and evaluate education is not only the need of discipline development but also the necessity of social development. Fuzzy evaluation extends the application scope of mathematics from deterministic field to fuzzy field, that is, from precise phenomenon to fuzzy phenomenon. The application of fuzzy evaluation in education injects vitality into the development and evaluation of education, improves the quality and efficiency of education evaluation, and reflects some basic characteristics and complexities of education. It points out a new research direction for the development of educational evaluation (Table 1).

## Figures and Tables

**Table 1:** Possible mappings.

Falling in love	Bicycling
People who fall in love	Rider
Active action	Pedal plate

## Data Availability

The experimental data used to support the findings of this study are available from the corresponding author upon request.

## References

[1] van de Laar T., de Regt H. (2008). Special section: is cognitive science changing its mind?. *Theory & Psychology*.

[2] Tomasello M. (2008). *Origins of Human Communication*.

[3] Kristeva J., Gong S., Wang D. (2018). Universal linguistics and the “poor linguist”. *Contemporary Rhetoric*.

[4] Gong Y. (2018). Also on why metaphor is possible. *Contemporary Rhetoric*.

[5] Zhu F., Zhang C., Zheng Z., Farouk A. (2021). Practical network coding technologies and softwarization in wireless networks. *IEEE Internet of Things Journal*.

[6] Yanxia F. (2015). The embodied philosophical paradigm of metaphor study. *Philosophical Dynamics*.

[7] Chen W., Huang J. (2012). Embodiment, metaphorical understanding and emotional initiation. *Zhejiang Social Sciences*.

[8] Xu S., He X., Li P. (2003). Self-organizing process neural networks and their applications. *Computer Research and Development*.

[9] Larsen-freeman D. (1997). Chaos/complexity science and second language acquisition. *Applied Linguistics*.

[10] Musolff A. (2006). Metaphor scenarios in public discourse. *Metaphor and Symbol*.

[11] Musolff A. (2012). The study of metaphor as part of critical discourse analysis. *Critical Discourse Studies*.

[12] Group P. (2007). MIP: a method for identifying metaphorically used words in discourse. *Metaphor and Symbol*.

[13] RitchieRitchie L. D., Cameron L. (2014). Open hearts or smoke and mirrors: metaphorical framing and frame conflicts in a public meeting. *Metaphor and Symbol*.

[14] Semino E., DemjénKoller V. (2014). ’’‘’’Good’ and ‘bad’ deaths: n. *Discourse Studies*.

[15] Semino E., Zsófia D., Jane D. (2016). *An Integrated Approach to Metaphor and Framing in Cognition, Discourse, and Practice, with an Application to Metaphors for Cancer*.

[16] Farouk A., Batle J., Elhoseny M. (2018). Robust general N user authentication scheme in a centralized quantum communication network via generalized GHZ states. *Frontiers of Physics*.

[17] Lesh R. (2006). Modeling students modeling abilities: the teaching and learning of complex systems in education. *The Journal of the Learning Sciences*.

[18] Rortveit G., Schei E., Strand R. (2004). Complex systems and human complexity in medicine. *Complexus*.

[19] Dickmeyer N. (1989). Metaphor, model, and theory in education research. *Teachers College Record: The Voice of Scholarship in Education*.

[20] Lankford B. A., Craven J. (2020). Rapid games designing; constructing a dynamic metaphor to explore complex systems and abstract concepts. *Sustainability*.

[21] Freeman D. L., Cameron L. (2008). Research methodology on language development from a complex systems perspective. *The Modern Language Journal*.

[22] Koithan M., Bell I. R., Niemeyer K., Pincus D. (2012). A complex systems science perspective for whole systems of complementary and alternative medicine research. *Forschende Komplementärmedizin/Research in Complementary Medicine*.

[23] Guliyev F. A. O. (2021). Metaphor as an object of the synergy paradigm study. *Linguistics and Culture Review*.

[24] Diez Roux A. V. (2011). Complex systems thinking and current impasses in health disparities research. *American Journal of Public Health*.

[25] Remondino M. (2003). Agent based process simulation and metaphors based approach for enterprise and social modeling. *ABS*.

